# The Good, the Bad, the Clunky: Improving the Use of Administrative Data for Research

**DOI:** 10.23889/ijpds.v4i1.587

**Published:** 2018-12-04

**Authors:** Kerina Helen Jones, Sharon Heys, Karen S Tingay, Paul Jackson, Chris Dibben

**Affiliations:** 1 Population Data Science, Swansea University Medical School, Swansea University, Swansea, UK, SA2 8PP; 2 University of Essex, Colchester CO4 3SQ; 3 Edinburgh University, Edinburgh, UK, EH8 9XP

**Keywords:** Administrative data research, data access, data linkage

## Abstract

**Introduction:**

Administrative data arising via the operation of public service delivery systems hold great benefits for citizens and society by enabling new research questions to be addressed, providing they can be made available in a safe, socially acceptable way. In recognition of this potential, the UK Administrative Data Research Network was established in 2013 to enable new research for public benefit. However, there are considerable challenges to be overcome for effective data use, and many of these are common to administrative data enterprises in general. Using this network as a practical case study, we set out to explore the issues and propose how to share the ‘good’, suggest solutions to the ‘bad’, and improve the ‘clunky’ issues, to lead to improvements in administrative data use.

**Methods:**

A qualitative survey representing the data use pathway was carried out across the network, followed by a workshop to discuss the summarised findings and make further suggestions. This led to a set of recommendations to inform the development of an action plan for implementation.

**Results:**

The survey respondents (N=27) and workshop participants (N=95) comprised multi disciplinary staff from across the network. The responses were summarised by consensus of three researchers and grouped into six areas: A) Data acquisition pathway; B) Approval processes; C) Controls on access & disclosure; D) Data and metadata; E) Researcher support; and F) Data reuse & retention, leading to an embedded set of 18 recommendations. Key developments promoted by this study were the development of themed research partnerships to progress data acquisition, and a policy of data retention and reuse for research.

**Conclusions:**

The network has broken new ground in using administrative data for research. This study informed the development of an evidence-based action plan to address many challenges in the effective use of administrative data. It represents a practical worked example, and the learning is widely relevant to enterprises working with administrative data across the world.

## Introduction

Administrative data can be defined as data arising via the operation of public service delivery systems, that is, information collected primarily for administrative (not research) purposes. These data are collected by a range of organisations such as government departments, local authorities, education establishments, social housing provision, and health & social care providers for the purposes of registration, transaction and record keeping, usually during the delivery of a service [1,2]. The structure of data of most interest to quantitative researchers is unit record data of the whole population of interest, with inter-disciplinary, longitudinal and cross-sectional attributes. Such data hold a vast array of potential benefits for citizens and society providing they can be made available for research in a safe, socially acceptable way. The datasets represent a rich resource of individual-level records that can be used to answer important questions about issues that impact on people’s lives, their health and wellbeing, as well as societal issues and resourcing for public services. They are particularly powerful when they can be brought together and linked at the individual level. Across the world, there are many successful infrastructural developments enabling data-intensive research using linkable administrative data, but with the exception of some of the Scandinavian countries [3], the majority of data used to date has been derived from health records, with far less availability of data across the fuller scope of administrative data, alluded to above.

In recognition of this imbalance, the UK Economic and Social Research Council (ESRC) commissioned an Administrative Data Taskforce (ADT) to advise on improving access to, and linkage, between government administrative data for research and policy purposes. The resulting report acknowledged the great untapped potential in UK administrative data, and this, supported by a positive UK government response, led to the establishment of the Administrative Data Research Network (ADRN) in 2013 [4,5]. The ADRN comprised four Administrative Data Research Centres (ADRCs), one in each of the four UK nations, and an Administrative Data Service with a co ordination role [6]. The total investment was £42M, divided among the components for a 5-year period.

From the outset, the aim of the ADRN was to enable new research using administrative data for public benefit and significant headway has been made. A range of datasets is available through the ADRCs, and a selection of almost 100 case studies have been published on the ADRN website to showcase the breadth of research that has been conducted. These include topics such as: special needs educational provision; the dynamics of disability benefits; developing measures of social capital; relationships between health and homelessness; anti-depressant prescribing; improving home energy efficiency; finding work on leaving prison; sociodemographic patterns in active commuting; and the effect of airport noise on mental health [7].

Each ADRC operates a data repository model within its jurisdiction and makes data accessible for research in safe settings. Models of data access vary with the perceived sensitivity of the data. For example, ADRC-Wales is built upon the models in place for the Secure Anonymised Information Linkage (SAIL) Databank. SAIL is a national data safe haven of de identified person-based data about the population of Wales, with a rich array of datasets including, general practice, in patient and out-patient hospital data, screening services, cancer registry, education, and birth & death records [8,9]. These data can be linked together at the individual level and made available in anonymous form to accredited researchers via the SAIL Gateway. The Gateway is an ISO 27001 certified data sharing and analysis platform, surrounded by a robust, proportionate data governance regime with privacy-by-design. It enables researchers to access the data for which they have approval, on their own desktop anywhere in the world [10]. Where ADRC data are deemed to be particularly sensitive, a researcher may be required to work within an on-site dedicated safe room with additional security measures and surveillance as further safeguards. As well as safe rooms within ADRCs, the ESRC has extended on-site data access by commissioning a network of micro safe settings (known as SafePods) based within higher education institutions and other organisations across the UK [11,12].

However, there are still many challenges to address, much scope for improvement, and an appetite to move this forward. The ADRN is not unique in this regard, as highlighted in a recent report on data availability and use in Australia [13]. Since the ADRCs work to common specifications as part of the ADRN, together they formed an ideal case study of the challenges in working with administrative data. **The aim** of this independent research study was to explore good practice, barriers and bottlenecks in the effective use of administrative data, and to propose how to share the ‘good’, solve the ‘bad’ and improve the ‘clunky’ issues, to lead to improvements in how administrative data are used for research. With its focus on the data use pathway across the ADRN, this study represents a partial evaluation of the implementation of the ADT report [4]. Because there are many common challenges, and the widely growing interest in the reuse of routinely-collected data, this paper will be of value to any administrative data enterprise.

## Methods

This paper is focused on developing a better understanding of the issues around effective administrative data use using the ADRN as a case study. As such, it does no’t explicitly cover the whole gamut of associated work areas, such as technology, infrastructure, communications and public engagement, etc., though they may be implicitly included. Information for the study was gathered from across the ADRN in two stages. The first was a qualitative on-line survey with a free-text response format and the second was a workshop to discuss the summarised findings and make further suggestions for addressing the identified challenges. The survey asked about the ADRN data use pathway, which is defined as the route from the identification of potential datasets through to data archiving. The 18 separate steps are shown in Appendix 1. An ‘other topics’ category was included at the end of the survey to allow respondents to identify additional points. The survey was not framed as individual questions, but as a request, that for each step in the pathway, respondents would provide their views under the headings of good, bad and clunky issues, with corresponding suggestions for sharing, solving and improving them, respectively. Respondents were asked to provide their job role, but were not asked to provide personal information.

There was no obligation to comment on all topics and an additional ‘Other’ category was included to allow respondents to comment on any topics not included. The survey was produced in Google Forms and distributed across the ADRN by the main communications team, and it was open for a 3-week period between 21st April and 12th May 2017. As the survey was cascaded across the network, the response rate cannot be calculated. The responses (N=27) were reviewed, themed and summarised manually by consensus of three researchers.

The processed responses to the survey were categorised into 6 areas to facilitate discussion in the workshop. These were:

A)Data acquisition pathway(Qs 1-3)B)Approval processes(Qs 4-6)C)Controls on access & disclosure(Qs 7-9)D)Data and metadata(Qs 10-13)E)Researcher support(Qs 14-16)F)Data reuse & retention(Qs 17-18)

The workshop was designed to further develop thinking on the issues emerging from the survey and was held at an ADRN general meeting in Edinburgh on 31^st^ May 2017 (N=95 in 10 groups of 9-10 people). It was facilitated by KHJ, with assistance from SH & KT. Summarised findings on each area were randomly provided to two separate groups of delegates for discussion, except for A) and F). This was because there were already dedicated ADRN workstreams on the data acquisition pathway (led by PJ) and data reuse & retention (led by CD). Each of these areas was allocated to a single group and the discussion facilitated by the respective workstream lead. The groups of delegates were asked to review the summaries and to choose one or more issues to discuss and provide feedback on how the ADRN could best progress in that area, by practical measures and influence. Each group wrote their comments on paper and gave verbal feedback in a plenary session at the end of the session. The written comments and notes (taken by two of the authors, SH & KT) on the verbal feedback were transcribed and used to create a synthesis of main points and suggested actions by area (by KHJ), which were then reviewed for consensus by three researchers (KHJ, SH & KT). The findings and recommendations were shared with the workstream leads for A) data acquisition pathway and F) data reuse & retention for feedback, and to include summary plans for these areas of work in the study report to the network directors. The report was provided to the directors in September 2017 and, following their deliberations, they produced an action plan for implementation. This was shared with the lead author (in January 2018) and the complete study was presented at the Administrative Data Research conference in June 2018 [14].

## Results & Discussion

### Profile of participants

All four ADRCs and the ADS were represented in the 27 responses to the survey: three from ADRC Northern Ireland; six from ADRC Scotland; two from ADRC Wales; five from ADRC England; five from the ADS; and six respondents didn’t give their affiliation. The job roles included were management, research and support, senior academics, researchers, directors, project managers, and staff involved in user services, teaching, public engagement, communications, office administration, data indexing, and data negotiation. At the workshop (N=95) there were 15 people associated with ADRC Northern Ireland, 33 from ADRC Scotland, 13 from ADRC Wales, 14 from ADRC England and 20 from the ADS. The roles profile was: 22 from management, 48 from research and 25 from support roles.

### Main findings by area

The main ‘good, bad and clunky’ findings from the survey and the workshop discussions, along with actions suggested by the participants for sharing the good, solving the bad and improving the clunky issues, are summarise by area in Table 1. Sometimes the same point may occur in different categories, for reasons such as variations in practice and respondent experience (e.g. in D), linkage quality is seen as good and bad).

The additional survey question (‘other topics’) allowed respondents to make further points. Where possible, these have been incorporated into the areas above. As noted, the aim of this study was on elucidating issues to lead to improvements in data use, but two important cross-cutting themes were identified. These were: the need for better, more regular, communications across the network; and to acknowledge that public engagement needs more attention, with a suggestion for more events with the public and wider stakeholders.

Respondents said:

‘Public opinion can be useful to the ADRN acquisition agenda if people could be convinced that de identified linked data could provide an advantage in the delivery of their services or in the efficiency of the economy.’

‘Courting public opinion should always include a full and clear inclusion of the privacy safeguards which then builds trust’

‘I think some of the bottlenecks are lack of awareness of or importance placed on public engagement’

‘Need buy-in from leadership beyond recognising public engagement as crucial to the set-up of the ADRN and moving beyond low-level engagement like events and newsletters to thinking deeper about how public engagement can enhance research and research impact and placing a duty upon researchers to get involved.’

### Recommendations

The combined findings of the survey and workshop provided a rich source of viewpoints and ideas for improvement. They were used by the researchers (KHJ, SH & KT) to develop a set of 18 recommendations across the six areas. These are shown below:

- **Data acquisition pathway**
  1) A regularly updated and well-signposted information resource on datasets, identifying the data custodian and providing metadata.
  2) A more streamlined data acquisition process, including a tracker on progress.
  3) A standardised process for data provider permissions, with greater clarity on who the decision makers are, shared protocols and agreed target timelines.
- **Approval processes**
  4) Greater transparency of regulatory approval processes and consistent advice to researchers, with more alignment between regulatory and network approval processes to avoid duplication.
  5) Proportionate peer-review, inclusive of wider stakeholders, with the network accepting the funder’s peer review where relevant.
  6) Clearer information on the circumstances where consent to link datasets is/is not needed, and on consent specificity, to inform discussions with data providers at the outset.
- **Controls on access and disclosure**
  7) Network approvals process to include cross-national UK studies, with greater use of remote access facilities and more safe rooms/pods.
  8) Greater clarity on what constitutes a disclosure risk and transparency on the Statistical Disclosure Control (SDC) measures applied to the data before being accessed for research.
  9) Harmonised training for those who check results for release, and opportunities for dialogue between checker and researcher.
- **Data and metadata**
  10) Recognising that data formats will vary, there is need for standardised documentation and versioning.
  11) Greater levels of communication with data providers to share data quality reports and gain information on data item provenance.
  12) Standardised data linkage quality reports and guidance for researchers.
  13) Consistency between metadata and dataset content through dialogue with data providers and a standardised metadata catalogue.
- **Researcher support**
  14) Consistent researcher support to be available across the network, and through the project life cycle, with agreed response times.
  15) More training for researchers on data manipulation and analysis to address the skills gap.
  16) Up-to-date tools and software provided in a timely way.
- **Data reuse and retention**
  17) Projects to be clustered into themes and all data to be reusable within a safe setting.
  18) Clearly defined stewardship of retained data, and an asset registration number for each dataset.

**Table 1: Good, bad and clunky findings and suggested actions:** ^1^This entails groupings of partners taking forward the creation of new linked datasets [14]. ^2^The UK Digital Economy Act (2017) extends the opportunity for data sharing by government departments http://www.legislation.gov.uk/ukpga/2017/30/contents/enacted. ^3^The 5Ps plan is a set of principles devised by Paul Jackson (ADS Strategic Data Negotiator) and stands for personality, prospectus, pathway, partnership and planning the service. ^4^This is where data are brought together for research, but are deleted when no longer required for the study. This, and theming are expanded upon in the discussion on existing workstreams.

**A) Data acquisition pathway**	

**Good, bad and clunky issues** Good: presence of knowledgeable research support officers, and good relationships with data providers with streamlined permission processes for data acquisition. Bad: difficulty in identifying data custodians, lack of information on data acquisition progress, and inconsistent processes between organisations. Clunky: lack of complete dataset documentation, uncertainty about identity of decision-makers, and differing interpretations of legislation and regulations.	**Suggested actions** Increasing dialogue between data providers, ADRCs and researchers to promote the value of data sharing for research, and to provide assurance of risk mitigation. Placing the focus on research themes^1^ rather than on individual government departments. Adopting the principles of the Digital Economy Act^2^ and implementing the 5 Ps plan, plus the 6th P for ‘define the product’^3^ .

**B) Approval processes**	

**Good, bad and clunky issues** Good: regulatory and peer review approval processes seen to be working well, despite their occasional complexity, plus addressing the regulatory issues with data providers at earliest stage. Bad: diverse interpretations of legislation and regulations, duplication of processes and over-reliance on participant consent. Clunky: approval processes not transparent to researchers, uncertainty on when data custodian approval is required, and over-concern about disclosure risks due to record linkage.	**Suggested actions** Streamlining network approvals processes and allowing researchers to attend the peer review panel to address queries upfront. Documenting and sharing experiences of going through approval processes to identify common issues and inform others. Providing case studies to illustrate consent requirements.

**C) Controls on access and disclosure**	

**Good, bad and clunky issues** Good: flexibility in safe settings for data access, and working towards common principles for mitigating disclosure at data access and results stages. Bad: approvals being by nation and not across boundaries, too few safe settings, and lack of transparency on what disclosure control measures have been applied. Clunky: having to travel to safe rooms/pods, overly restrictive disclosure control, and not grasping that unique is not equal to identification.	**Suggested actions** Building confidence in network trustworthiness by including the 6th P – product - in the ‘5Ps’ plan which would be attractive to data providers, and including this in the network prospectus. Developing a set of flexible principles enabling researchers to self-check their proposed outputs with reference to experts. Providing common training for checkers and training for researchers.

**D) Data and metadata**	

**Good, bad and clunky issues** Good: compatible/standard data formats, good metadata, and linkage quality reports. Bad: data not matching the data dictionaries, metadata limited or not provided, and lack of information on linkage quality. Clunky: lack of clarity on who should solve data formatting issues, and insufficient feedback to data providers on data quality.	**Suggested actions** Increasing dialogue with data providers to emphasise the value of good quality datasets and accurate metadata. Documenting and sharing solutions to tricky issues. Acknowledging intellectual effort needed, creating a persisted asset listing for datasets, and a dataset citation index to track dataset usage.

**E) Researcher support**	

**Good, bad and clunky issues** Good: research support officers not being tied up in administration, variety of training course and analysis tools. Bad: lack of data analysis and manipulation skills among researchers, and this not coming to light early enough to provide them with support. Clunky: inconsistency in support across the network, and in access to up-to-date and specialist software.	**Suggested actions** Clarifying expectations and documenting the roles of researcher support staff. Increasing connections with outside networks for mutual support ideas, training, funding and collaboration. Conveying to funders that greater timing flexibility is needed for data-intensive research to allow for unknown delays in data delivery to researchers.

**F) Data reuse**	

**Good, bad and clunky issues** Good: clustering projects into themes, and reusing data to save on extraction time and effort. Bad: create-and-destroy^4^ data use model is seen as a waste of resources, with too short a period before the data are deleted. Clunky: the need for data provider permission before data use, and researchers wishing to keep data for their exclusive use.	**Suggested actions** Encouraging data reuse and requiring good reasons before supporting a project unwilling to allow reuse. Moving away from create-and-destroy, and building transparency into data retention models, including the levels of control data providers wish to retain in the reuse of their data, with class approval for similar projects. Building awareness among data providers and funders of the value of data retention, with due regard to risk mitigation.

### Existing workstreams

The workstream leads for A) data acquisition pathway and F) data reuse and retention provided their feedback on the study, and a summary of the developing plans for these areas of work to be included in the report to the network directors. The study findings were received as a welcome contribution to the review and revision of ADRN policies, and it was noted that the results strongly reinforced the need, and provided practical evidence for the case, to move away from a create-and-destroy model to one where data are retained for reuse.

During 2016-17 the ADRN had formed a task team to review network policy on data retention and destruction. The network had originally adopted the conservative position that linked data should only be available to single projects and then, when these projects had completed their work, the data destroyed. Whilst this was seen as a sensible initial position to take, the potential benefits of moving towards the reuse of data for suitability qualified research teams were becoming very apparent. The task team carried out a wide ranging review: analysing the legal considerations of a potential move to the reuse of data for research purposes; exploring the public’s views on data reuse through public panel meetings and reviews of the literature; reviewing practice in other countries; and exploring data reuse in the context of the ADRN core principles. The review established that there were no insurmountable barriers to a change in data reuse policy. As a result of the review and the findings of this study, and following discussions with senior UK government officials, network policy was changed to one of data reuse rather than create-and-destroy. These discussions were essential since much of the administrative data of interest is held in government departments. This change in policy will reduce duplication of effort and lead to greater data use for public benefit. It was acknowledged that that data reuse would be implemented at various different levels within the network depending on discussions with data controllers.

In order to advance work on the data acquisition pathway, there has been a greater focus on research themes, with groupings of partners with an interest in each theme taking forward the creation of new linked datasets [15]. These datasets are then made available for reuse in functionally anonymised form by researchers with the necessary approvals. Functional anonymization asserts that a holistic, contextual approach should be used to determine anonymisation, taking into account the data environment, not just the status of the dataset. This includes the presence of other data, the agents accessing the data, the data governance model, and the infrastructure in place [16]. The reuse of data in research themes will simplify the approval processes, as the departmental approvals needed to create the datasets are separate from the project approvals needed to use those data for research. It will also support consistency in SDC measures, the development of dataset metadata, and will streamline support provision to researchers, as teams may follow a particular theme and become expert in the associated datasets.

### Action plan

The ADRN directors developed an action plan for implementation, based on the recommendations of the report and in light of changes already in motion. In doing this they highlighted a key message in each of the six areas: A) to F), work underway and future actions. This is summarised in Appendix 2.

### Study in context

This unique study used the ADRN as a case study to uncover ‘good, bad and clunky’ points in using administrative data for research, and to suggest ways to highlight the good, solve the bad and improve on the clunky issues. As we were interested in improving data access and use for research, we chose to focus on topics along the data use pathway from data acquisition to data archiving. The establishment of the ADRN stemmed from the Administrative Data Taskforce report, and this study provides an in-practice evaluation of the adoption of the Taskforce recommendations for data use [4]. It is a practical example in that: it is based on a working case study; it sought the views of multidisciplinary network staff at all levels; it made recommendations to the network directors; and these were taken forward to implement improvements to promote data use. The inclusive approach empowered network staff to contribute to the direction the network should take, and provided the directors with a body of evidence on which to base their decisions.

This study has wide relevance since working with administrative data brings common challenges. These were highlighted in a recent review, namely, data generation, management, analysis, quality, access and linkage [1,17]; some of which we have addressed in this paper. Our findings also accord with those of an Australian Government report, which noted legal barriers, risk aversion among data providers, and hindrances to data access, amongst other important issues [13]. Furthermore, many of our recommendations correspond with those made in the OECD Expert Group report on International Collaboration on Microdata [18]. This shows that the experiences of the ADRN are not unique, and that the adoption of effective practice can be informed by international experience. As such, this paper presents an exemplar that can be used as a guide to other administrative data enterprises. This work will be of value across the rapidly growing field of Population Data Science, as the ‘science of data about people’, which encompasses work with all types of person-based data [19].

### Lessons learned

With its practical focus, this study presents valuable lessons to be learned in working with administrative data. We share these lessons for others working in administrative data enterprises, or setting out to do so. For data acquisition, we found that government departments need a clear programme of work that clarifies potential benefits on a wide scale. The themed approach to data acquisition is showing promise as a better paradigm than merely requesting datasets, as it has revealed to government departments more about the possibilities of research using administrative data. It is not enough talk about the value of data per se; there need to be more compelling arguments for the potential outcomes, including the return on investment from data provision. Approvals processes need to be both robust and proportionate so that they protect data providers and citizens, but are not a hindrance to research [20]. Where research is conducted across a network of organisations, standardised approval processes with equivalence will be beneficial to minimise duplication of effort. Similarly, standard training for researchers, consistent metadata, and common SDC should be in place, where possible, to promote compatibility and comparative research. Although some researchers are *au fait* with the complexities of working with administrative data, support for researchers is much needed, and requires dedicated resource with expertise in the provenance, format and quality of datasets. All these lessons reinforce the importance of reusing datasets, rather than operating a create-and-destroy model, to avoid wasted time and effort in discarding hard won research resources. Data reuse, of course, must be properly governed with trustworthy data stewardship.

Although our focus was not specifically on communications and public engagement, these are essential cross-cutting themes which we, and analogous enterprises, are wise to heed. At least for the ADRN, the development of research themes, and partnership groups to develop the associated datasets, have been key in implementing the action plan, and work is ongoing for further improvements. Good communication channels and on-going dialogue with the range of stakeholders, notably partnerships with data providers, are essential to avoid misunderstandings and duplication of effort. We highlight that effective communications and public engagement are essential for social licence in the use of person-based data [21]. The ESRC are committed to investing in administrative data enterprises, and have reorganised the ADRN into the Administrative Data Research Partnership (ADRP). This represents a major shift as the Partnership does not specifically include ADRC-England or the ADS, but creates a major role for the Office of National Statistics. It also places a greater focus on working closely with data providers to generate impact, and an extended public engagement programme since social licence is far more than bare legality [21].

There is also much further work to be done in relation to legal and ethical issues. Data from healthcare providers has long been legally provided to data centres by means of an NHS trusted third party to de identify the personal data: for example, the working arrangement between the NHS Wales Informatics Service and the SAIL Databank [8]. The introduction of the UK Digital Economy Act (2017) has been a positive step towards enabling greater availability of (non-health) administrative data, with its provisions for the use of trusted third parties for data anonymization [16]. The socially -acceptable, increased reuse of administrative data is greatly welcomed, not only to gain public benefits, but to avoid the massive harms and costs to society known to occur when data are not used [22]. The increased focus on stakeholder and public engagement will be paramount to addressing the issues and achieving these aims.

### Limitations

The main limitations of this study are that: some issues might have been missed since not everyone in the network completed the survey; response time was restricted to a three week window for practical reasons, so that the findings could be discussed at a pre-scheduled workshop; and the survey was only distributed to network staff and did not include wider views. Because survey respondents were not required to provide personal details, we cannot be sure that the views are representative of the whole network. However, the ADRCs operate on the same principal model, are subject to the same challenges, and we obtained responses from a range of job roles. As such, although not necessarily fully representative, we have no reason to believe the findings are invalid. Future work in due course, could be to evaluate progress since the implementation of the action plan, and to include wider stakeholders in the process. This study has not been able to address all the challenges and how they could be overcome. We propose there should be an evaluative research programme to guide investments in wider use of linked administrative for research by governments and by independent researchers.

## Conclusions

The ADRN has broken new ground in using administrative data for research in the UK. This innovative, practical study has revealed many good, bad and clunky issues along the data use pathway, has set out recommendations for evidence-based improvement, and shared the lessons learned. On reflection, the formation and development of the ADRN required new thinking and greater understanding of wider perspectives amongst the research community, data owners and the governance/oversight community. Consequently, we emphasise the central importance of effective stakeholder communications to enhance efficiency in data use. The conclusions reinforce many of the ADT recommendations, and inform others in the light of experience, to progress the effective use of administrative data. The findings and recommendations of this study are informing the work of the Administrative Data Research Partnership. Importantly, since many of the challenges are ubiquitous, this study is highly relevant generically, and as such, will be valuable to other enterprises working with a broad range of administrative data.

## Abbreviations

Administrative Data Research Centres (ADRC); the Administrative Data Research Network (ADRN); Administrative Data Service (ADS); Administrative Data Taskforce (ADT); Organisation for Economic Co-operation and Development (OECD); Secure Anonymised Information Linkage (SAIL); Statistical Disclosure Control (SDC)

## Appendix 1: The survey topics along the data use pathway

A brief description is shown alongside each step in the pathway. The pathway is not intended to be definitive, but is an approximation for the purposes of the survey.

**Table:** ^5^Please note that in the survey Q9 ‘Disclosure control in release of results’ followed ‘Availability of analysis tools’, as it was set out to approximate the order of the data use pathway. After the survey, the question responses were grouped into 6 areas, resulting in a slight change of order: placing Q9 into C) Controls on access and disclosure, since they are similar in topic. As the new order is used through the remainder of the paper to the recommendations, the question numbers in the table have been set out accordingly.

	Step	Description

1	Identifying potential datasets	Gaining awareness of datasets of interest, their locations and their data custodians
2	Acquiring datasets	Legal, technical and procedural processes for transferring datasets
3	Obtaining data provider permissions	The types of permissions required and how to apply
4	Regulatory approval processes	Navigating and securing lawful and ethical approvals
5	Peer-review approvals	The requirements of network and funder peer-review panels
6	Obtaining consent to link data	Understanding when consent to link is required and how to go about gaining it
7	Accessing data	The processes by which data are accessed
8	Disclosure control in data access	The measures applied to mitigate risk in data accessed for research
9^5^	Disclosure control in release of results	The measures applied to mitigate risk in results released for dissemination
10	Data formats	Dealing with differences in data formats and compatibility
11	Data quality	Issues of completeness and accuracy
12	Linkage quality	Reliability of the linkage process
13	Metadata	Dataset descriptors and documentation (for locating and using data)
14	Support available to researchers	How to provide effective support to researchers
15	Acquiring analysis skills	The range of skills needed for data querying and manipulation
16	Availability of analysis tools	Ensuring a range of tools are available to data users
17	Reuse of administrative data	Processes for enabling the reuse of data, as opposed to one-off uses
18	Data retention and archiving	Having a suitable process to retain and archive data beyond the project life-span.

## Appendix 2: ADRN action plan

A summary of the action plan developed by the ADRN directors as a result of this study.

**Table:** In terms of the other topics identified, i.e. the need for better communications across the ADRN and more attention to public engagement, a cross-network directors’ update was introduced, and the public engagement work of the ADRCs in Scotland and Wales was acknowledged, along with a need to extend the work.

Area		Key message	Actions

A)	Data acquisition pathway (recommendations 1-3)	Need for a more streamlined and definite process for data acquisition, with good information and data documentation	A programme of workshops involving a wide range of stakeholders was initiated in November 2017. Each workshop sought to agree on a research area and to develop datasets to match this. Four main themes were agreed upon: world of work; data for children; growing old; and productive society [14]. The themed partnerships aim to deliver a standardised process for data acquisition with more predictable timescales.
B)	Approval processes (recommendations 4-6)	Concern was raised over duplication within the approval processes and the need for clear guidance	The network peer-review approvals panel undertook a self-assessment exercise. The panel considered the benefits of researchers attending meetings and decided that follow-up outside meetings would be more effective. This approvals panel is independent of the ADRN and so makes decisions on its own operation. The themed partnership approach will meet the recommendations on clarifying consent, as theme partners will take on the role of licencing authorities, defining the conditions for reuse of the data they create.
C)	Controls on access and disclosure (recommendations 7-9)	Secure settings and disclosure control are valued within ADRN, and emphasis should be put on facilitating access cross-nationally	The series of stakeholder workshops, with a focus on research themes, is enabling the development of a more standardised process for data access to data with more predictable timescales. ADRC Scotland is leading on a programme to increase the availability of safe settings (safe pods) for accessing data. A common outcome from all the workshops is a focus on enriching data for longitudinal studies. Increased training in SDC is being planned.
D)	Data and metadata (recommendations 10-13)	Emphasis on documentation of data and good metadata is needed	The theme partners are working to develop datasets for each research theme. As the datasets are produced, they are documented and metadata is developed. Data quality reports will be shared with data providers and they will be fully involved in the testing of the dataset for use. From time to time, guides are commissioned to provide an overview of the data and its background, and datasets will be curated with persistent identifiers.
E)	Researcher support: (recommendations 14-16)	Need for more consistent support and communication with researchers	Researcher support staff are based at each ADRC and the teams are coordinated across the ADRN by the ADS. This is seen as a good service, however, it is recognised that each ADRC has its own local procedures which may be, at least partly, the cause of the identified inconsistencies. Research funders are being included in the themed workshops so they have fuller knowledge of timescales in working with administrative data, and the need to build in flexibility.
F)	Data reuse & retention: (recommendations 17-18)	The ADRN should move towards a reuse model	ADRN policy has been changed, moving away from create-and-destroy to data reuse for research. The themed approach concentrates on the delivery of curated datasets which are functionally anonymised and made available for reuse in research by accredited researchers. Projects have been clustered into themes and used as exemplars as part of discussions during acquisition and development of the datasets. Clearly defined stewardship of retained data will be needed, with an asset registration number for each dataset, and agreed arrangements for archiving.
